# 2D and 3D Bulk Materials for Environmental Remediation: Air Filtration and Oil/Water Separation

**DOI:** 10.3390/ma13245714

**Published:** 2020-12-15

**Authors:** Ha-Jin Lee, Won San Choi

**Affiliations:** 1 Western Seoul Center, Korea Basic Science Institute, 150 Bugahyun-ro, Seoudaemun-gu, Seoul 120-140, Korea; hajinlee@kbsi.re.kr; 2 Department of Chemical and Biological Engineering, Hanbat National University, 125 Dongseodaero, Yuseong-gu, Daejeon 305-719, Korea

**Keywords:** air filtration, oil/water separation, 2D and 3D bulk materials, environmental remediation

## Abstract

Air and water pollution pose an enormous threat to human health and ecosystems. In particular, particulate matter (PM) and oily wastewater can cause serious environmental and health concerns. Thus, controlling PM and oily wastewater has been a great challenge. Various techniques have been reported to effectively remove PM particles and purify oily wastewater. In this article, we provide a review of the recent advancements in air filtration and oil/water separation using two- and three-dimensional (2D and 3D) bulk materials. Our review covers the advantages, characteristics, limitations, and challenges of air filters and oil/water separators using 2D and 3D bulk materials. In each section, we present representative works in detail and describe the concepts, backgrounds, employed materials, fabrication methods, and characteristics of 2D and 3D bulk material-based air filters and oil/water separators. Finally, the challenges, technical problems, and future research directions are briefly discussed for each section.

## 1. Introduction

Air and water pollution have been growing and challenging concerns worldwide because of rapid civilization and industrialization. Air pollution from particulate matter (PM) has become one of the most severe global challenges in the 21st century. Particularly, PM has been a major national, environmental, and social issue in developing countries due to serious long-term effects on the ecosystem as well as on human health [1,2,3,4]. According to a report from the World Health Organization (WHO), more than 80% of the world’s population in 2018 was exposed to air quality levels that exceed WHO limits [5]. Worldwide air pollution tremendously endangers human health because of poisonous pollutants such as heavy metals, toxic gases, and microorganisms [6,7]. To protect the public from severe air pollution, air filters are highly necessary and have been intensively studied in recent years. Ideal air filters should have the ability to effectively remove PM particles while allowing air to pass through them easily. Conventional air filters have low specific surface areas and poor surface affinity for PM because air filters are usually made of micrometer-sized polyethylene (PE) and polypropylene (PP) fibers that insufficiently interact with PM. The materials employed for conventional air filters insufficiently interact with PM because conventional air filters are usually made of micrometer-sized polyethylene (PE) and polypropylene (PP) fibers. Conventional air filters have low specific surface areas and poor surface affinity for PM. To achieve an air filter with high performance, various materials have been developed, including synthetic polymers, natural polymers, nanocarbons, inorganics, and organic/inorganic hybrids [8,9,10].

Water pollution by oily wastewaters is another major national, environmental, and social issue. Since oily wastewater has been increasingly emitted by industry and daily life activities, it has become a severe global environmental concern and threat. Oil spill accidents frequently occur in the ocean as well as on land [11,12,13,14,15]. These spills occur during exploitation, transportation, utilization and storage [16]. Oily wastewater is also a major problem in many industries such as chemical plants, manufacturing industries, crude oil production, petroleum refineries, lubricant manufacturing, textile processing, and food production [17,18,19,20,21]. Conventional methods such as adsorption, sedimentation, gravity separation, electrocoagulation, and biological treatment have been widely used for oil/water separation [22,23,24,25,26]. However, these methods have limited satisfactory solutions because chemical and biological methods can often cause secondary pollution to the surroundings. Physical adsorption methods also have the disadvantages of low absorption capacity and rates for oil/water separation. To address this issue, a variety of oil/water separation materials and techniques have been proposed [27,28,29,30,31,32,33,34,35,36,37,38,39,40].

Nano/micromaterials are of interest in environmental remediation because of their high surface-to-volume ratio and uptake capacity, which leads to a higher adsorption or catalytic performance [41,42,43,44,45,46,47,48,49,50,51,52,53,54]. Although nano/micromaterials possess such fascinating characteristics, they have difficulties being used alone due to issues in mass production, high product cost, and unintended leakage. In particular, it has been recently reported that the unintended leakage of nano/micromaterials into ecosystems can cause a significant threat to the environment and human health [55,56]. Most of the reported nano/micromaterials are flow type and disperse in water or solvent during use, which become vulnerable to leakage during the process. Moreover, nanoparticles (NPs) tend to aggregate due to their high surface areas and strong dipole–dipole interactions. Thus, various types of NPs have been synthesized onto various matrices to prevent aggregation and increase the surface area of the NPs [57,58,59,60,61,62,63,64,65,66,67,68]. However, most of the reported matrices loaded with NPs are nano/micrometer-sized materials that still possess the aforementioned problems. Therefore, to improve the strength and compensate for the weakness of nano/micromaterials, 2D and 3D bulk materials loaded with nano/micromaterials have been reported for environmental remediation, including air filtration and oil/water separation. Various 2D and 3D bulk materials have been utilized for air filtration and oil/water separation (Table 1). Most 2D and 3D bulk materials are used as a single or combined type for both applications. Advanced air filters and oil/water separators based on typical 2D and 3D bulk materials will be discussed.

**Figure 1:**
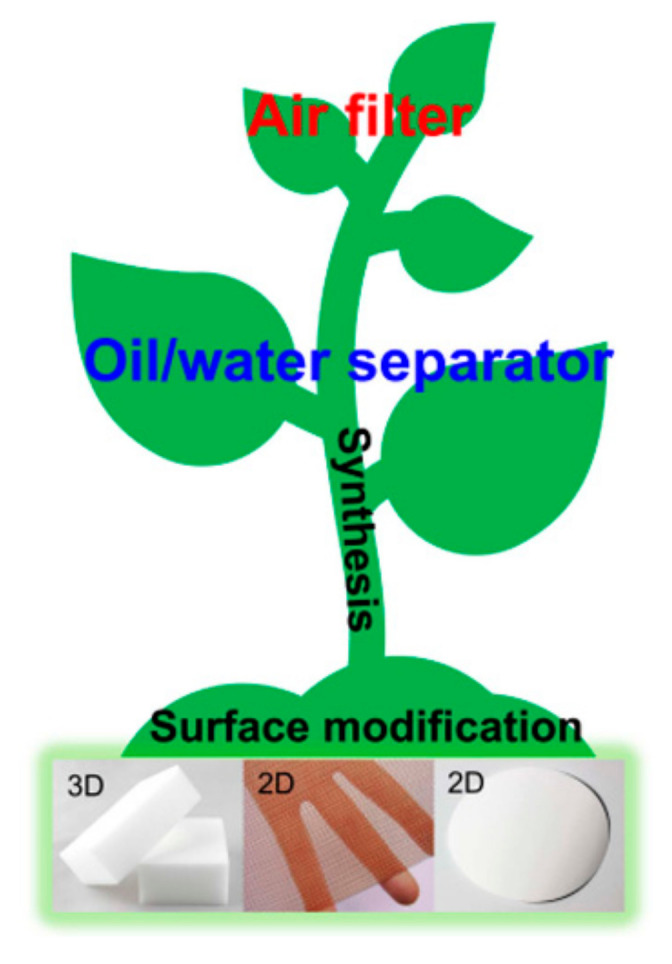
Schematic illustration for 2D and 3D bulk material-based environmental remediation.

Herein, we reviewed the recent advancements of 2D and 3D bulk materials and their applications in air filtration and oil/water separation (Figure 1). This review begins with an introduction of the significance and necessity of environmental remediation using bulk materials. Then, the background, classification, mechanism, characterization, and materials of air filters are presented (Part 1). The next part outlines oil/water separation processes using 2D and 3D bulk materials. This part contains the basic concepts, mechanisms, and materials of oil/water separation (Part 2). Finally, the current challenges in the fields of air filtration and oil/water separation using bulk materials are discussed.

## 2. Air Filtration (Part 1)

### 2.1. Basic Concept of PM

**Figure 2:**
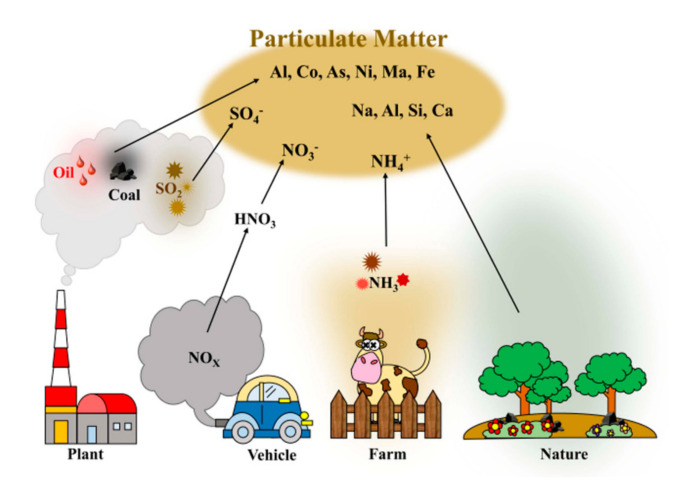
Schematic illustration for particulate matter (PM) generated from various sources.

Polluted air contains PM of various sizes, chemical vapors, viruses, bacteria, and so forth. PM poses a great threat to human health and ecosystems because it has serious effects on climate, solar illumination, air visibility, and ecosystems [69,70,71]. PM is constantly generated all over the world by nature and human activities from incomplete fossil fuel combustion, vehicle emissions, biomass burning, and industrial emissions (Figure 2) [72,73,74]. PM is a chemical mixture composed of components such as NO_3_^−^, SO_4_^−^, Cl^−^, organic and elemental carbon, and heavy metals [75,76,77,78,79,80,81]. PM can also contain various other toxic gaseous molecules such as methane (CH_4_), nitrogen dioxide (NO_2_), carbon monoxide (CO), and formaldehyde (HCHO). PM can be classified into several types by its aerodynamic diameters. PM_10_, PM_2.5_, and PM_0.3_ refer to particles with aerodynamic diameters of less than 10, 2.5, and 0.3 μm, respectively. Since PM_2.5_ particles can stay in the air for a long time and spread farther under atmospheric circulation, PM_2.5_ is more toxic than PM_10_ to humans. In particular, PM_0.3_ can penetrate human bronchi/blood vessels and travel through the circulatory system, resulting in respiratory/cardiovascular diseases and even cancer [1,2,3,4]. Thus, morbidity and mortality are greatly increased with long-term exposure to PM_2.5_.

### 2.2. Classification of Air Filters

Air filters have been widely used in various applications, including residential buildings, automotive industries, clean rooms, and hospitals. Air filters can be classified into three main types: particulate, chemical, and antimicrobial filters. Particulate air filters remove PMs of different sizes. Chemical air filters contain activated carbon materials that can capture gas molecules. Antimicrobial air filters include antimicrobial nanoparticles such as Ag, ZnO, or TiO_2_ that can kill bacteria upon contact. Among the aforementioned air filters, particulate air filters that can remove PM are mainly discussed in the following sections because particulate air filters account for a large proportion of air filters.

### 2.3. Particulate Capturing Mechanisms

Particulate air filters with high efficiency particulate air (HEPA) performances are generally used for the removal of PM from air. The filtration mechanisms of commercial air filters can be defined by their inertial, diffusion, interception, and gravity effects [82,83,84,85]. First, PM particles deviate from the air flow streamline when the flow changes and strikes the filtration materials, leading to an inertial impact. Thus, the inertial capture efficiency is influenced by the air velocity as a function of the Reynolds number. The larger the PM size, the greater the inertia and the higher the inertia efficiency. Second, a diffusion mechanism occurs when submicrometer PMs are captured by the filtration materials. Diffusion enables the submicrometer PMs to move in a random manner, resulting in deviation from the original air flow streamline [83,84]. The smaller the submicrometer PM size is, the more intense the random movement is. Theoretical calculations have indicated that the diffusion capture efficiency becomes greater than 80% when the particle size is less than 0.1 μm. Third, an interception capture mechanism occurs when PMs with a diameter larger than the pore size of the filters move with air flow. The filtration process is dominated by interception when the PMs are blocked by filters with pore diameters smaller than the PM size. Fourth, PM can also be captured by the assistance of gravity when air flow perpendicular to the ground occurs [83,84]. The aforementioned traditional air filters can achieve a high removal efficiency by using thicker filters or small pores, while their pressure drops remarkably increase. Recently, novel filtration mechanisms such as chemical or electrical forces have been reported and applied to capture PM particles [82]. PM particles possess many functional groups with high polarity, such as -SO_3_H, -NO_3_, -C-O, and -C-N [84]. Thus, the air filters with a high dipole moment can effectively capture PM particles by stronger dipole–dipole/induced-dipole and electrostatic interactions [84,85]. The electrostatic interactions are further enhanced to capture the PM particles if an electrical field is applied to the filter material, leading to an increase in removal efficiency [86,87].

### 2.4. Characterization of PM Filtration

Particulate air filtration is generally defined by three terms: removal efficiency (RE), pressure drop, and quality factor.

#### 2.4.1. Removal Efficiency and Pressure Drop

By comparing the PM concentrations before and after filtration, the RE can be calculated according to the following equation:

RE (%) = (C_0_ − C_1_)/C_0_ × 100% (1)

where C_0_ (µg m^−3^) and C_1_ (µg m^−3^) refer to the PM concentrations before and after filtration, respectively [84]. The pressure drop is the air flow resistance presented by the air filter. The pressure drop is calculated by comparing the upstream and downstream air flow pressures according to the following equation:

ΔP (Pa) = P_up_ − P_down_ (2)

#### 2.4.2. Quality Factor (QF)

The quality factor (QF) is calculated by taking both ΔP (Pa) and RE (%) into consideration, which is used to evaluate the overall performance of air filters:

QF = −ln(1 − RE)/ΔP(3)

where ΔP and RE refer to the pressure drop and removal efficiency, respectively [82,83,88]. A higher quality factor indicates better air filter performance. The QF shows great improvement if the removal efficiency increases and pressure drop decreases. If the air filter structure is more complicated or hierarchical to make the pore size smaller, the removal efficiency and pressure drop will be greatly increased. Thus, there is a trade-off relationship between these two properties [89,90]. The air flow rate is another important parameter used to evaluate the performance of air filters. Both the pressure drop and removal efficiency are functions of the air velocity. The pressure drop will increase, but the removal efficiency will decrease if the air velocity increases. In other words, the air velocity is directly and closely linked to RE and ΔP. However, since the QF does not reflect the air velocity, Choi et al. proposed a modified QF (m-QF) that can reflect the air velocity factor to exactly evaluate the overall performance of air filters, where V is the air velocity [82]:

m-QF = −ln(1 − RE)V/ΔP(4)

### 2.5. Air Filter Materials

In recent years, various material-based air filters, such as synthetic polymers, natural polymers, carbon-based materials, and other materials, have been proposed for the removal of PM and VOCs (volatile organic compound). These emerging materials, which have shown desirable characteristics for PM and VOC-laden air purification, are discussed in the following sections.

#### 2.5.1. Synthetic Polymer-Based Materials

Since polar materials with higher dipole moments can have stronger dipole–dipole and induced-dipole intermolecular interactions to capture PM particles [84,91,92], various polymers with higher dipole moments, such as polyacrylonitrile (PAN), polymethyl methacrylate (PMMA), polyvinylpyrrolidone (PVP), and Nylon-66, have been used to prepare various air filters [93]. Cui’s group fabricated an air filter composed of PAN nanofibers by electrospinning method [84]. PM particles were captured by the nanofiber surfaces. The newly generated PM moved to the existing PM particles to form larger particles. Cui’s group also fabricated PI (polyimide)-based air filters for the removal of PM particles. The filters showed high PM removal efficiency (>99.5%) at a high temperature for exhaust gas purification [94]. Li et al. reported nanofiber/net air filters composed of poly(vinylidene fluoride) (PVDF) for effective PM removal [95]. The Steiner structure of air filters was formed by the synergistic effect of electrical and hydrophobic interactions, achieving a removal efficiency of 99.98%. Liu et al. fabricated washable hydrophilic and hydrophobic bilayer composite filters [96]. A nanofiber composite filter is composed of superhydrophobic PMMA/polydimethylsiloxane (PDMS) fibers to block moisture transfer and superhydrophilic chitosan (CS) fibers to remove PM. Transparent nanofiber composite filters showed an outstanding PM removal performance such as high RE and low pressure drop even at high humidity. Ding’s group prepared PVDF fiber-based air filters by electrospinning, which produced negative ions, effectively boosting the immune system [97]. A stronger surface potential was obtained due to the formation of more negative ions, resulting in a more enhanced PM removal ability. Many high-polarity hydrophilic air filters have been proposed for capturing PM. As discussed above, hydrophilic and hydrophobic polymers can be used for air filter materials. However, relatively little attention has been focused on hydrophobic materials and their roles. Thus, the study of hydrophobic materials is necessary for hybrid air filters.

#### 2.5.2. Natural Polymer-Based Materials

##### Cellulose

Cellulose, consisting of hundreds and sometimes even thousands of carbon, hydrogen, and oxygen atoms, is the most abundant polymer in nature. Since cellulose is the main component of the plant cell wall, it can be easily derived from a variety of sources in nature. Cellulose has been extensively studied for wastewater treatment [98,99], hydrogels and aerogels [100,101], and fiber reinforcing materials [102,103,104]. Recently, researchers have focused on cellulose materials for air filtration applications due to their mechanical properties and hydrophilicity.

**Figure 3:**
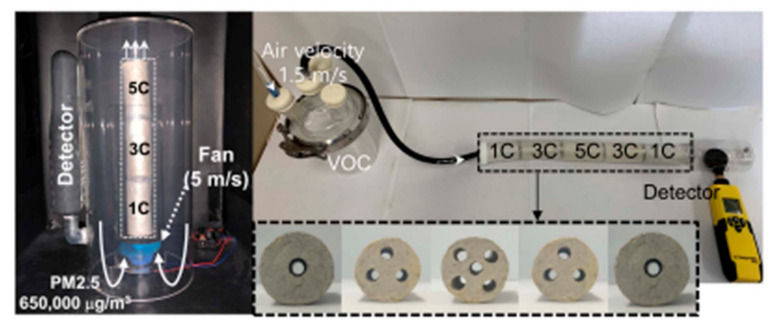
(**Left**) An image of the operation of an air cleaner equipped with CHAF-1C/3C/5C for the removal of PM_2.5_. (**Right**) Images of an air filtration system equipped with CHAF-1C/3C/5C/3C/1C and single cylindrical hollow air filters (CHAFs) (CHAF-1C, 3C, and 5C) for the removal of VOCs (HCHO). Reproduced with permission from [100]. Copyright 2020 American Chemical Society.

A study conducted by Choi et al. reported a wastepaper-based cylindrical hollow air filter (CHAF) module for the removal of PM_10_, PM_2.5_ and HCHO [100]. They reported that removal efficiency and pressure drop could be increased and decreased by CHAFs that are connected in series and parallel, respectively (Figure 3). PM_2.5_ removal efficiency and pressure drop are further increased and decreased by combinations of CHAFs connected in series and parallel, respectively. A CHAF-based miniature air cleaner was demonstrated to remove a high amount of PM_2.5_ (5 L) at high concentrations of over 650,000 μg/m^3^ and a high flow rate of 5 m/s with a high removal efficiency (99.24–96.88%) and low pressure drop (31–34 Pa) for up to 270 cycles. Ogi et al. synthesized a cellulose/PVP nanofiber composite for the removal of PM_0.3–0.5_ particles [101]. They reported that the removal efficiency of 87% for PM_0.3–0.5_ and a pressure drop of 17 Pa were achieved by cellulose-based air filters. The filtration efficiency was not as high as that of a commercial HEPA filter (99.97%), while a very low pressure drop was achieved compared to that of commercial HEPA filters (ΔP = 150–200 Pa). Nicosia et al. reported electrospun cellulose acetate-based air filters for high-efficiency and heat-resistant air filtration applications [102]. They studied the filtration performance and the pressure drop of the filters as a function of the weight of the nanofiber layer. The cellulose acetate-based air filters showed very good filtration efficiencies for PM sizes below 50 nm and above 300 nm. However, the removal efficiency dropped from approximately 95% to approximately 60% for PM_0.1_. The aforementioned studies demonstrate that cellulose, with inexpensive and biodegradable characteristics, possesses great potential for air filtration.

##### Chitosan

**Figure 4:**
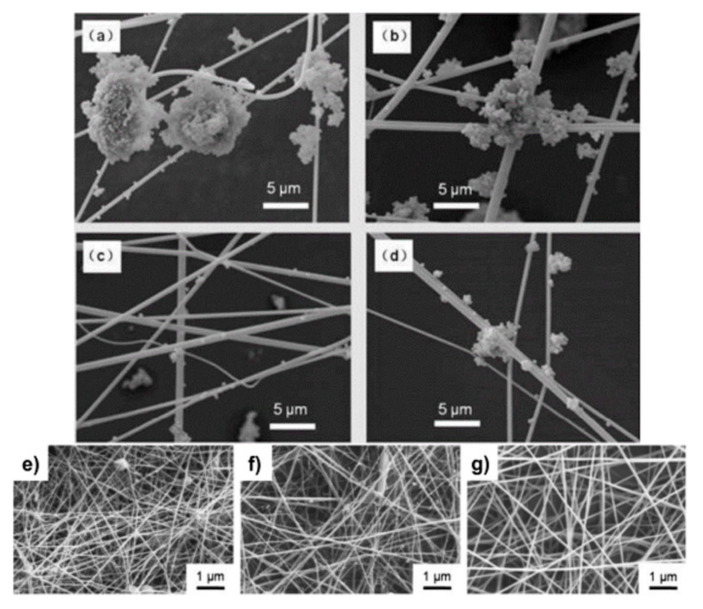
SEM images of e-spun (**a**) chitosan, (**b**) polyacrylonitrile (PAN), (**c**) polyvinylpyrrolidone (PVP), and (**d**) PS (Polystyrene) nanofibers in the polluted air. SEM images of 1.33 wt% HMW (high molecular weight) chitosan:PEO (polyethylene oxide) (90:10) fibers spun on spunbonded polypropylene (PP) using (**e**) 75% acetic acid (AA), (**f**) 90% AA, and (**g**) 75% AA (2 mM). Reproduced with permission from [104]. Copyright 2017 The Royal Society of Chemistry.

Chitosan is a sugar that can be easily obtained from the hard-outer skeleton of shellfish, shrimp, and other crustacean shells. Chitosan has been extensively used for air filter materials for the following reasons: (i) chitosan can be easily polarized due to its strong polarity because it is composed of randomly distributed b-(1-4)-linked D-glucosamine and N-acetyl-D-glucosamine, benefiting the effective capture of PM; (ii) chitosan possesses antimicrobial properties to inhibit the growth of bacteria, fungi, and yeast [103]; (iii) chitosan also has nontoxic and biodegradable characteristics because it is a kind of polysaccharide obtained from chitin. These characteristics make chitosan a good selection for air filter materials. Long et al. developed chitosan-based air filters using an in situ electrospinning method (Figure 4a–d) [104].

An outstanding filtration performance was achieved by the strong electrostatic attraction of the chitosan-based air filters. The outstanding performance of the chitosan-based air filters can be explained as follows: (i) chitosan can easily generate additional charges for enhancing the interaction between the chitosan air filters and PM particles; (ii) positively charged chitosan amine groups can easily interact with negatively charged PM groups, such as SO_4_^2−^, NO_3_^−^ and Cl^−^, to enhance the interactions between chitosan air filters and PM particles. These factors greatly increase the removal efficiency of PM by chitosan-based air filters. Since chitosan possesses good antimicrobial properties, researchers have also focused on chitosan-based air filters with antimicrobial properties. Kit et al. investigated chitosan/PEO-based air filters with varying fiber diameters (Figure 4e–g) [104]. The PM removal efficiency of the chitosan/PEO air filters was decreased by increasing the fiber diameter of the filter. The antimicrobial characteristics of the chitosan/PEO air filters was also investigated. The positively charged amine groups present in chitosan can interact with the negatively charged groups in the cell wall of *E. coli* bacteria, deactivating and killing the bacteria. The chitosan/PEO air filters showed a 3-log reduction in an *E. coli* colony forming unit after 6 h of contact. He et al. synthesized chitosan/PVA composite air filters with antibacterial properties for PM capture using a phase separation technique [105]. The structure transformed from a honeycomb to a sponge-like morphology by varying the chitosan concentration. This air filter, with a thickness of 37 μm, showed a removal efficiency of 95.59% and a pressure drop of 633.5 Pa. This air filter also possessed excellent antibacterial properties for *E. coli* and *S. aureus*. These studies indicate that chitosan, an abundant natural material, has great potential for developing air filters with high performance and antimicrobial properties.

#### 2.5.3. Carbon-Based Materials

Many researchers have used nanocarbons for the synthesis or preparation of air filters owing to their high specific surface areas and excellent adsorption capacity [106,107,108]. Carbon nanotubes (CNTs) can provide several additional advantages for air filters. First, CNTs have little effect on the air streamline due to the smaller diameter, leading to a decreased pressure drop [109]. Second, CNTs are highly suitable for air filtration because of their good mechanical properties, such as tensile modulus (<2 TPa) and strength (100–200 GPa) [110]. CNTs have been employed as a critical component of air filters due to their fascinating characteristics. Yildiz and Bradford investigated a CNT-based composite air filter [111]. They used a combination of multiple layers of aligned CNT nanosheets and wound them onto a PP fabric mat to improve the PM_0.3_ removal performance to 99% because the PP fabrics could effectively contribute to trapping the CNTs. A study by Li et al. developed a type of multilayered CNT/quartz fiber (QF) filter using a chemical vapor deposition (CVD) process to grow CNTs on QF [112]. The CNT/QF filters showed a slightly increased pressure drop but significantly increased the removal efficiency. The CNT/QF filters also showed high hydrophobicity, making them suitable for use in a high humidity environment. Halonen et al. reported a simple way to prepare CNT composite air filters by organizing aligned CNTs onto macroscopic films for PM filtration with a removal efficiency of >99% [113].

**Figure 5:**
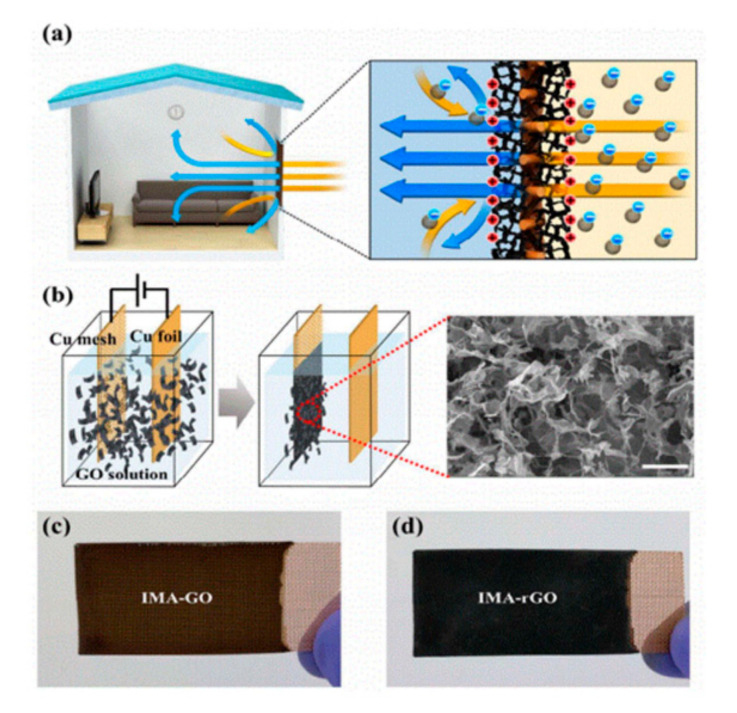
PM_2.5_ removal mechanism, fabrication process, and the structure of reduced graphene oxide (GO)-based air filter: (**a**) the schematic illustration of PM removal by a reduced GO-based air filter through electrostatic attraction force; (**b**) the schematic illustration of the fabrication process for a reduced GO-based air filter. SEM image of the GO-coated surface (scale bar: 50 μm). Optical image of (**c**) GO-coated Cu mesh and (**d**) reduced GO-coated Cu mesh after (**c**) before and (**d**) after the thermal reduction process. Reproduced with permission from [115]. Copyright 2018 The Royal Society of Chemistry.

Much effort has also been devoted to graphene oxide (GO) aerogels for the application of air filters due to their large specific surface area, rich oxygen-containing groups, and low density. Dai et al. synthesized stable GO aerogels with a honeycomb structure by a freeze-casting method with the assistance of tourmaline NPs [114]. The GO composite aerogels exhibited excellent removal efficiencies due to their superior adsorption characteristics and steady large pore structures. In another work, Kim et al. developed reduced GO (RGO)-based air filters possessing large pores with a small pressure drop (Figure 5) [115].

RGO formed on both sides of the Cu meshes. The RGO/Cu mesh filter could capture PM particles and prevent them from entering the indoor space through air flow. Simultaneously, the inside of the RGO/Cu mesh filter could purify household PM particles. The quality factor of the RGO/Cu mesh filter was almost twice the best reported in the literature. Repeated regeneration and reuse with little loss of efficiency demonstrated the robustness of the RGO/Cu mesh filter.

#### 2.5.4. Inorganic-Based Materials

Researchers have utilized various types of inorganic materials for various applications due to their chemical, mechanical, and thermal stabilities even under severe conditions [116,117,118,119]. Chen et al. synthesized free-standing γ-alumina nanofibrous air filters with excellent removal performance against aerosol particles [120]. Even after heat treatment at 700 °C, this air filter maintained excellent removal performance with a removal efficiency of 99.85% and a pressure drop of 239 Pa, which suggests that the γ-alumina air filters have potential for PM filtration at high temperatures. In another study, Ding et al. employed sol–gel and electrospinning methods to fabricate SiO_2_ nanofiber air filters with excellent thermal stability [121]. The air filters exhibited significant removal ability for NaCl aerosols with a high removal efficiency of 99.99% and a low pressure drop of 163 Pa. The air filters also exhibited high mechanical properties, including tensile strength (5.5 MPa), tensile modulus (114 MPa), and strain at break (23.5%). This group also developed a SiO_2_/polyetherimide nanofibrous air filter for the removal of PM [122]. The filters exhibited excellent superhydrophobic properties, endowing them with better self-cleaning characteristics compared to commercial PP filters. In addition, the filters still showed a removal efficiency of 99.99% and a pressure drop of 61 Pa even after treatment at 200 °C for 30 min. These studies suggest that inorganic materials can also be used for developing air filters with high performance levels under severe conditions.

#### 2.5.5. Organic/Inorganic Hybrid-Based Materials

**Figure 6:**
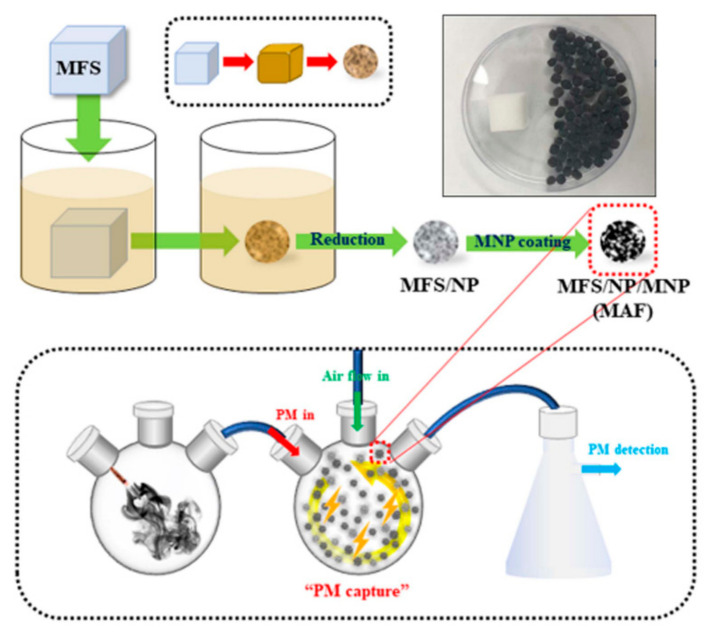
A schematic illustration of movable air filter (MAF) formation and a novel air filtration system in which MAFs are vigorously moved or rotated for the removal of PM particles. Inset: an melamine formaldehyde sponge (MFS) (1.12 × 1.12 × 1.12 cm^3^) and 100 MAFs. Reproduced with permission from [82]. Copyright 2019 The Royal Society of Chemistry.

Organic/inorganic hybrid materials based on synthetic polymers are discussed here. Synthetic polymers can be decorated with specific additives to enhance their filtration performance. For example, Choi et al. reported a lottery draw machine-inspired movable air filter (MAF) system for the first time (Figure 6) [82]. Spherical MAFs can be rotated within the glass chamber to generate a high electric field. They synthesized a millimeter-sized spherical MAF by the chemical and physical etching of a cubic melamine formaldehyde (MF) sponge. The glass chamber and metal NPs on MAFs became positively and negatively charged, respectively, by collisions between the glass chamber and MAFs. Thus, an electric field between the glass chamber and the MAFs can be built. The PM particles within the glass chamber could be effectively removed through electrostatic attraction. Another study by Ikegami et al. demonstrated gold/zirconium oxide (Au/ZrO_2_)-based air filters with photocatalytic and thermal catalytic characteristics [123]. They investigated the catalytic effects of Au/ZrO_2_-coated poly(ethylene terephthalate) (PET) air filters on the removal of CO gases and HCHO. Their results indicated the conversion of HCHO and CO to H_2_O and CO_2_ with rates of 90 and 83%, respectively. They also confirmed that the Au/ZrO_2_/PET air filters have high CO and HCHO decomposition ability into H_2_O and CO_2_. Metal NPs can be used not only to enhance the filtration performance but also to endow new functionality. Many researchers have employed various types of metal NPs to realize antimicrobial properties. Antimicrobial characteristics can be realized by treating or mixing fibrous filters with conventional antimicrobial materials such as Ag, TiO_2_, ZnO, and CuO NPs [124,125,126,127]. However, these NPs can cause irreversible damage to the human beings and the eco-system. Thus, additional studies are necessary to address safety issues for future study. The aforementioned studies demonstrate that synthetic polymers can be used as matrices and functionalized with various metal NPs to enhance the removal performance of PMs and VOCs.

## 3. Oil/Water Separation (Part 2)

### 3.1. Basic Concept of Oil/Water Separation

#### 3.1.1. Oil Penetration or Absorption

The lotus leaf has a superhydrophobic characteristic. A spherical water droplet can be formed on a lotus leaf. A water droplet on the leaf surface will easily roll off and remove the contaminant when the lotus leaf is slightly shaken, which is called the “self-cleaning” effect [128,129,130]. A lotus leaf shows a hydrophobic dual surface structure composed of micro/nanostructures, lifting the water droplet because of the air cushion trapped underneath the water droplet [131,132,133,134,135]. This is called the “Cassie wetting state” [132,133]. In other words, a hierarchical dual structure with a low surface energy is indispensable for superhydrophobicity, which endows lotus leaves with excellent superhydrophobicity.

#### 3.1.2. Water Penetration or Absorption

A fish scale has underwater superoleophobicity, allowing fish to freely swim in oily wastewater without contamination. This underwater oil repellency is based on the superoleophobicity of the fish scales in water [136]. The surface of the fish scale is coated with dual structures consisting of nano/micrometer-sized structures. In addition, the chemical composition of the fish scales consists of hydrophilic calcium phosphate and protein. The hierarchical rough surface morphology and the hydrophilic chemical composition endow the fish scales with superhydrophilicity and underwater superoleophobicity [129,136,137]. The hierarchical rough surface morphology of fish scales is wet by water because water can be trapped in such structures, forming a thin water cushion layer onto the surface of the fish scales. When an underwater oil droplet encounters the fish scale, the oil droplet will be repelled by the trapped water cushion. The combination of the hierarchical rough surface morphology and hydrophilic chemical composition allows the underwater Cassie wetting state, endowing the fish scale with superhydrophilicity, underwater superoleophobicity, and oil repellency [135,136].

### 3.2. Mechanisms of Oil/Water Separation

**Figure 7:**
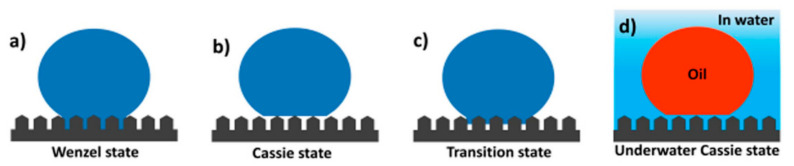
Four types of contact models of a droplet on solid substrates. (**a**–**c**) Liquid droplet on the rough surface at different wetting states: (**a**) Wenzel state; (**b**) Cassie state; (**c**) transition state; and (**d**) underwater Cassie state—oil droplet on the hydrophilic rough surface in water.

Water contact angles (WCAs) smaller or larger than 90° are classified as “hydrophilic or hydrophobic”, respectively. WCAs smaller or larger than 10° or 150° are classified as “superhydrophilic or superhydrophobic”, respectively. When the substrate is tilted and the droplet starts to roll off, the tilted angle is called the sliding angle (SA). The CA explains the static behavior of the surface, and the SA explains the dynamic property of a droplet on the surface, determining the overall surface wettability of the materials [135,138,139]. Thus, surface wettability is evaluated by testing the water or oil CA and SA. The wettability behavior of a droplet on a flat surface can be described by the Young wetting model [135,140]. However, this model is not suitable for rough surfaces because rough surfaces have a great effect on the wettability of materials in addition to their chemical composition [130]. The wettability behavior of a droplet on a rough surface can be explained by the Wenzel state, transition state, and Cassie state (Figure 7) [135,139,141,142]. In the Wenzel state, a liquid droplet can fill the vacancies of hierarchical rough structures, and then the droplet wets the rough surface (Figure 7a) [143]. Thus, the rough surfaces show high adhesion behavior to a droplet at the Wenzel states. However, when the droplet has difficulty filling the vacancies of the rough structures and the surface repels the droplet, the droplet can stay on top of the rough surface with the assistance of air cushions trapped beneath the droplet, which is known as the Cassie state (Figure 7b). Thus, the rough surfaces show low adhesion behavior to a droplet at Cassie states. In the transition state, the droplet partially fills the vacancies of the rough surface (Figure 7c) [129,144,145,146,147,148,149,150,151,152]. The trapped air cushion can minimize the contact area between the rough surface and the droplet in the Cassie state, resulting in excellent water repellency. This Cassie mechanism can be extended to an underwater oil droplet onto the surface (solid/water/oil phases) (Figure 7d).

When a hydrophilic substrate with a rough surface is dipped in water, the rough surface can be completely wetted by water. If an oil droplet is placed onto the prewetted surface, a trapped water cushion within the rough surface will repel the oil droplet. The oil droplet just sits on the rough surface, which is considered “the underwater Cassie state” [135,153,154,155,156,157,158,159,160]. The repulsive force between the trapped water cushion and oil droplet endows the substrate with the oil repellent property. Thus, only oil can penetrate through the superhydrophobic filters as soon as an oil/water mixture encounters the superhydrophobic filters. However, water is not able to pass through superhydrophobic filters [161]. When the superhydrophobic absorbents encounter an oil/water mixture, they selectively absorb oil and repel water based on the repulsive force between the trapped air cushion and water. These superhydrophobic absorbents also absorb heavy oils under the water when they are immersed into an oil/water mixture [162,163]. Superhydrophilic absorbents prewetted with water can selectively absorb water and repel oil under the oil, based on the repulsive force between the trapped water cushion and oil [162].

### 3.3. Materials for Oil/Water Separation

There are two types of materials for oil/water separation: filtration and absorption materials. Most filtration materials, such as meshes, fabrics, and membranes, have a higher separation capacity (flux) than absorption materials and are suitable for high separation efficiency [164,165,166,167]. Thus, filtration materials have been extensively used in industrial settings. Filtration materials are less practical than absorption materials in cases of spill accidents, because oils should be rapidly removed in situ. Since absorption materials like 3D porous materials can simply be placed on contaminated sites in cases of spill accidents and will selectively remove oil or water, they do not suffer from these problems [35,38,39]. Furthermore, no expensive or specialized equipment is necessary for the removal of oil or water. We discuss here the recent progress in oil/water separation techniques based on filtration and absorption materials.

#### 3.3.1. Filtration-Based Materials

There are two commonly used filters for oil/water separation: superhydrophobic–superoleophilic and underwater superoleophobic filters [168,169,170,171,172]. For superhydrophobic–superoleophilic filters, only oil penetrates through the filters driven by superoleophilicity and gravity force as soon as a mixture of a solution of oil and water is poured onto the filters. However, water is not able to pass through the filters and stays on the mesh due to the superhydrophobicity of the filters [173,174,175,176,177]. As a result, oil is removed, leaving only water. For underwater superoleophobic filters, these filters usually possess superhydrophilicity (or hydrophilicity) in air and superoleophobicity in water. Thus, when the oil/water mixture is poured onto the underwater superoleophobic filter that is previously wetted by water, the water can pass through the filter due to the superhydrophilicity of the filter. However, oil is repelled by the prewetted filter due to underwater superoleophobicity [178,179,180,181,182]. As a result, water is removed, leaving only oil.

##### Mesh-Based Materials

**Figure 8:**
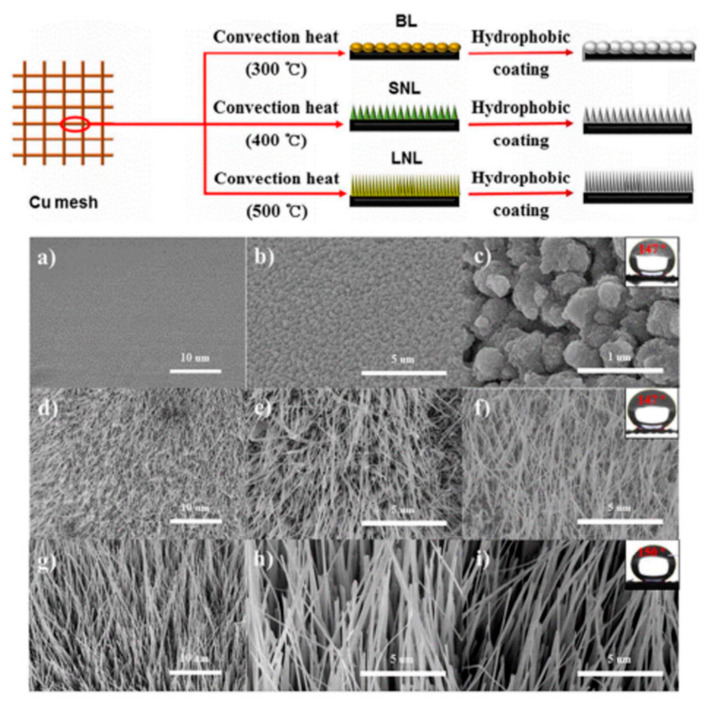
(**Top**) The schematic illustration of the hierarchically oxidized copper meshes formed by heat treatments. (**Bottom**) SEM images of the hierarchically oxidized copper meshes (**a**,**b**,**d**,**e**,**g**,**h**) before and (**c**,**f**,**i**) after a hydrophobic coating was applied: (**a**,**b**) bumpy-like (BL)-, (**d**,**e**) short needle-like (NL)-, and (**g**,**h**) long NL-Cu meshes synthesized at reaction temperatures of (**a**,**b**) 300 °C, (**d**,**e**) 400 °C, and (**g**,**h**) 500 °C, respectively. Reproduced with permission from [163]. Copyright 2017 American Chemical Society.

Functionalized meshes with superwettability have been of increasing interest to researchers seeking to separate oils from oil/water mixtures. Meshes have been ideal candidates for widespread use in real-life industrial applications because of their high separation efficiency and rate, simplicity, and low cost. In particular, metal meshes such as Cu and stainless steel meshes have been intensively used for oil/water separation because of their high mechanical properties. The most common metal meshes are copper oxides (CuO or Cu(OH)_2_) grown from a pristine Cu mesh. The morphology of these oxidized Cu meshes can be controlled by varying the preparation conditions. The oxidized Cu meshes are generally prepared by etching Cu meshes with NaOH, HCl, HNO_3_, or ammonium persulfate to yield a mesh covered with CuO or Cu(OH)_2_ nano/microwires [150,183]. Song et al. also fabricated stainless steel (SS) meshes possessing hierarchical surface morphology by immersing SS mesh in a solution of CuCl_2_ and HCl followed by a deposition of stearic acid [175]. The hydrophobic SS mesh exhibited excellent oil separation efficiencies of 94% and high filtrate purities of 99 wt% for oils with various viscosities. A chemical etching procedure is used to prepare superhydrophobic Cu meshes with rough structures because of simple and inexpensive methods. However, most Cu meshes with rough surfaces have been synthesized using toxic chemicals, including strong acids/bases and oxidants for synthesizing [161,184,185]. A convection heat treatment approach was proposed for replacing a previous method using toxic chemicals [163]. They synthesized hierarchically oxidized Cu meshes with various surface morphologies. Since this method is a simple and green route that does not involve toxic chemicals, it can replace existing chemical oxidation methods (Figure 8) [163]. A simple heat treatment of Cu meshes resulted in the formation of three types of hierarchically oxidized Cu meshes (Figure 8, top). By varying the reaction temperatures, the needle length of the Cu meshes could be controlled (Figure 8, bottom). After a hydrophobic coating of the oxidized Cu meshes, three types of Cu meshes exhibited excellent separation efficiencies of over 95% up to 20 separation cycles.

**Figure 9:**
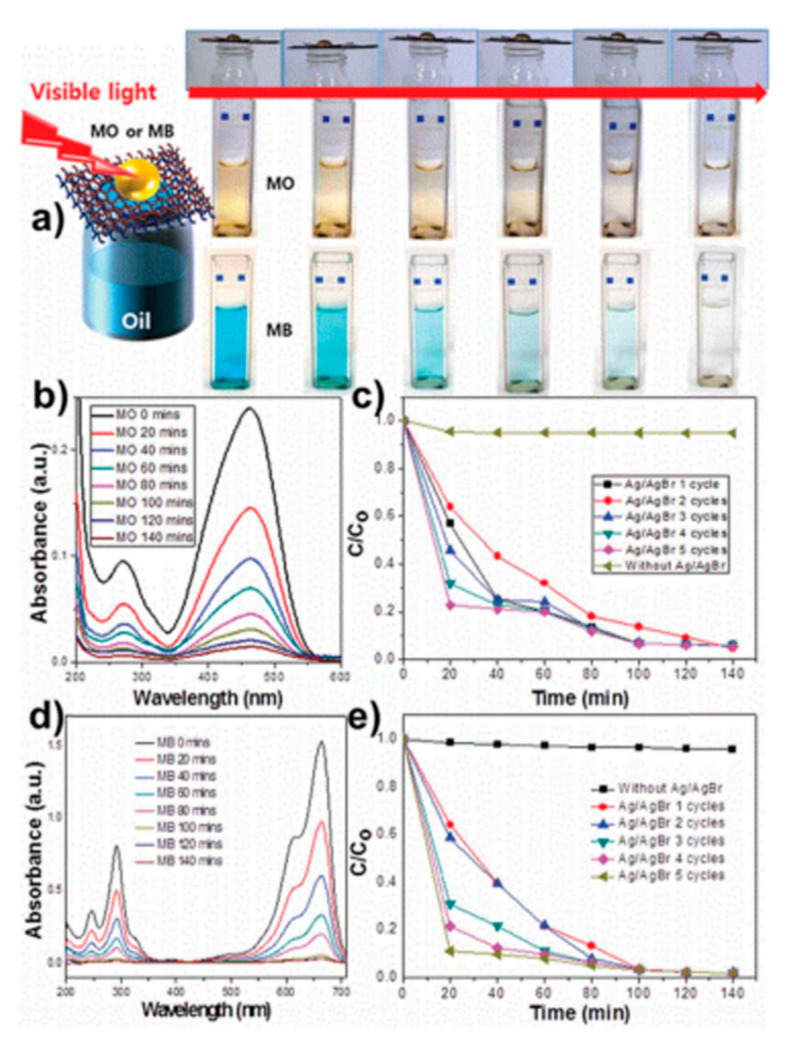
(**a**) Photocatalytic decomposition process demonstrating the purification of methylene orange (MO) and methylene blue (MB). Double-layer Cu meshes consisting of a photocatalytic mesh (Cu mesh/Pdop/Ag/AgBr) and a superhydrophobic mesh (Cu mesh/1H,1H,2H,2H-perfluorooctyltriethoxysilane (PFTOS)) were used for the test. The degradations of (**b**) MO and (**d**) MB catalyzed by a photocatalytic mesh under visible-light irradiation. The reuse test data of a photocatalytic mesh under visible-light irradiation for (**c**) MO and (**e**) MB up to 5 cycles. Reproduced with permission from [167]. Copyright 2016 The Royal Society of Chemistry.

Polymers can be used to functionalize metal meshes to create the wetting properties necessary for oil/water separation. Cationic or anionic polyelectrolytes have been utilized to synthesize superhydrophilic meshes. Meshes were coated with three types of block copolymers [186,187,188]. Surface modifications with polyelectrolytes resulted in meshes with superhydrophilic and underwater superoleophobic characteristics for oil/water separation. Polyacrylamide (PAM) hydrogels can also be used to prepare meshes possessing superhydrophilic and underwater superoleophobic characteristics [189]. A PAM-modified mesh can separate a range of oils, such as crude oil, gasoline, diesel, and vegetable oil, from oil/water mixtures. A superhydrophobic mesh can also be prepared by special interactions between polydopamine (Pdop) and octadecylamine (ODA) [190]. Pdop was coated on a Cu mesh (Cu mesh/Pdop). Spherical Pdop particles were generated at the surface of the Cu mesh/Pdop by the heat treatment of the Cu mesh/Pdop. The heat-treated Cu mesh/Pdop could be also used as a superhydrophilic mesh before the hydrophobic coating. After the ODA coating on the Cu mesh/Pdop, hierarchical ODA crystals were formed through the interactions between Pdop and ODA. The large number of Pdop particles induced the formation of hierarchical ODA crystal structures on the Cu mesh/Pdop. The possibility of the photo-induced decomposition of aqueous pollutants after oil/water separation was also studied using a polymer-modified mesh (Figure 9) [167]. Pdop and Ag/AgBr were coated onto an oxidized Cu mesh (O-Cu mesh) with a rough surface. The photocatalytic mesh was overlapped on the superhydrophobic mesh for a dual purpose of aqueous pollutant purification after oil/water separation (Figure 9a). The superhydrophobic mesh was prepared by coating 1H,1H,2H,2H-perfluorooctyltriethoxysilane (PFTOS) on the oxidized Cu mesh. The upper Cu mesh was coated with Ag/AgBr photocatalysts, resulting in the formation of a hydrophilic mesh (O-Cu mesh/Pdop/Ag/AgBr), while the lower mesh was superhydrophobic (O-Cu mesh/PFTOS). Hexane was quickly passed through upper and lower Cu meshes into a beaker below, while an aqueous methylene orange (MO) or methylene blue (MB) solution remained on top of the Cu meshes. Upon the visible light irradiation, the color of the MO or MB solution gradually faded because of the catalytic decomposition reaction between MO (or MB) and Ag/AgBr on the upper Cu mesh (Figure 9a). The UV–vis absorbance of the MO or MB solution remarkably decreased as a function of increasing irradiation time, demonstrating the decomposition of MO or MB (Figure 9b–e).

##### Fabric-Based Materials

Although metallic meshes have been used as promising templates for oil/water separation, metallic meshes have some disadvantages, such as easy corrosion from oxygen, heavy weight, and high cost. Since fabrics do not corrode and are characterized by their light weight, flexibility, and low cost, fabric-based materials could be also considered good candidates for oil/water separation. Superhydrophobic/superoleophilic and superoleophobic/superhydrophilic fabrics have been proposed for selective oil/water separation [191,192,193]. Zhou et al. reported polyaniline and fluorinated alkylsilane-coated cotton fabric using a facile CVD method [194]. They controlled the growth of nano/microinorganic crystals on fabrics to tune the surface roughness of fabric fibers. The cotton fabric exhibited a high separation efficiency of 97.8% due to superhydrophobicity with a WCA of 156° and superoleophilicity with an oil CA of 0°. A superhydrophobic and superoleophilic PE fabric was also developed by a growth of Si nanostructures onto the fabric by the CVD of trichloromethylsilane [195]. The PE fabric showed a water repellent property, which was applied for a filtration membrane-based oil/water separation. Wang et al. developed robust superhydrophobic/superoleophilic fabrics decorated with metal oxides and metallic nanocrystals by the in situ growth method [196]. They prepared various multiscale rough surfaces along with special wettability by coating with metal NPs. They also tuned the surface wettability by controlling the nucleation and growth of nanocrystals. A layered double hydroxide (LDH)-functionalized superhydrophobic/superoleophilic fabric was developed by coating commercial fabric with LDH microcrystals and low surface energy molecules [197]. These fabrics, with high separation efficiency (>97%), were used in the application of oil/water separation as well as selective oil absorption. Wang et al. fabricated fly ash-coated durably superhydrophobic fabric for oil/water separation [198]. The fly ash-coated fabrics showed a superhydrophobicity with a WCA of 152° due to the hierarchical surface morphology. These fabrics could separate oil/water mixtures with high separation efficiencies (97.3%). Moreover, these fabrics maintained their wettability characteristics under severe environments such as acidic, alkaline, UV irradiation, and high ionic strength conditions. The fly ash-coated fabrics still exhibited high separation efficiency up to 16 cycles and stable wettability.

**Figure 10:**
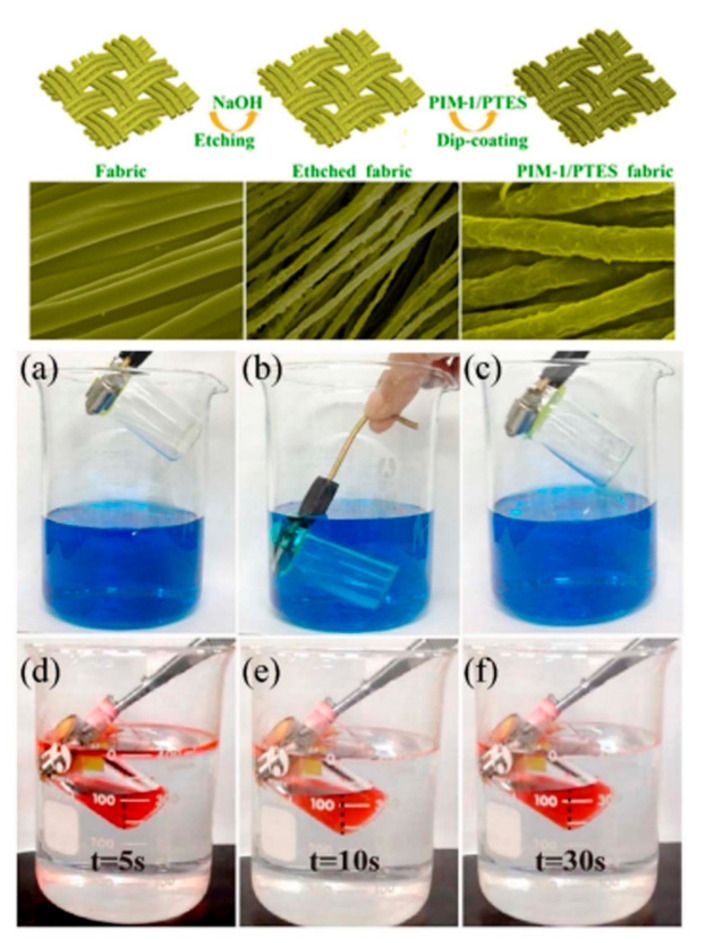
(**Top**) Schematic illustration of the superhydrophobic fabrics fabricated by etching and dip-coating process. (**Bottom**) Images showing oil–water separation and collection by a superhydrophobic fabric-equipped device. Images showing (**a**–**c**) no water collection and (**d**–**f**) hexadecane oil collection. Reproduced with permission from [202]. Copyright 2019 American Chemical Society.

Several groups have utilized a dip-coating method to endow fabrics with superhydrophobic/superoleophilic and superhydrophilic/superoleophobic properties. This dip-coating method is a simple way to prepare fabrics with special wettability. Zhang et al. employed a dip-coating method to prepare superhydrophobic cotton fabrics containing ZnO-NPs and PS on the surface of cotton fabric [199]. Moreover, these fabrics showed excellent wettability properties for the oil/water separation with WCA over 153°. Another superhydrophobic cotton fabric was also prepared by dipping the bare fabrics into a TiO_2_ solution followed by coating with stearic acid to generate a dual structure onto the surface of the fabrics [200]. Li et al. proposed a one-step dip-coating method using a mixture of PDMS and SiO_2_ dispersion to fabricate superhydrophobic fabric and utilized it for oil/water separation [201]. Both dip-coating and etching methods can be simultaneously applied to pristine fabrics to create superhydrophobic fabrics. Zhang et al. fabricated superhydrophobic/superoleophilic polyethylene terephthalate (PET) fabric possessing a dual surface morphology through the etching fibers with alkali and followed by dip-coating the PET fabric into a solution of fluorinated alkylsilane (Figure 10) [202]. The pristine PET fabric, consisting of microscale fibers with smooth surfaces, was etched by NaOH, resulting in enhanced roughness and reduced fiber diameters (Figure 10, top). After coating the polymer and fluorinated alkylsilane onto the etched PET fabric, the resulting fabric was mounted on a glass bottle and fabricated as an oil skimmer container (Figure 10, bottom). Oil/water separation and collection processes were performed by superhydrophobic fabric-capped device. This device showed a stable separation efficiency as high as 99% even after 30 cycles. Similarly, superhydrophobic fabrics with rough surface structures were fabricated by the alkali etching of fibers, followed by hydrophobic coating with mercapto silanes [203]. The superhydrophobic fabrics exhibited excellent chemical stability against various chemicals, such as acid, base, salt, acetone, and toluene and were utilized for oil/water separation.

##### Fiber-Based Materials

**Figure 11:**
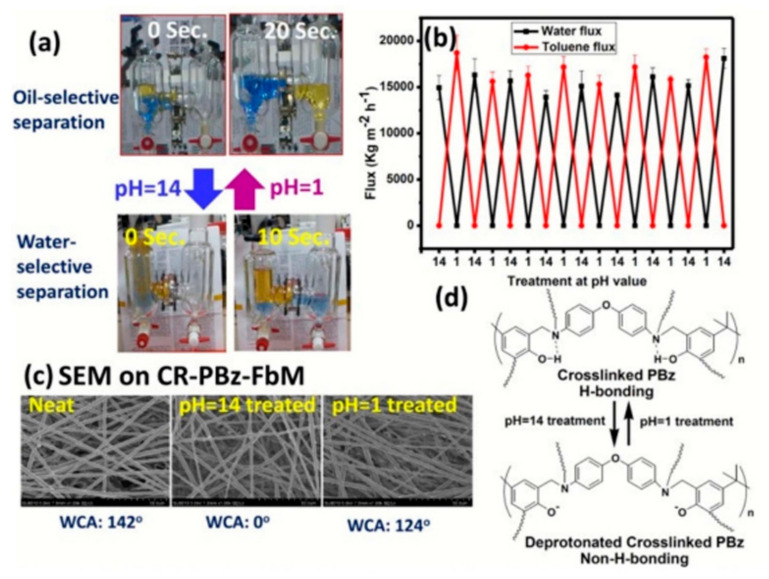
(**a**) pH-induced switchable membrane in oil/water separation; (**b**) flux data of the membrane at different pH values; (**c**) SEM images of the membrane at different pH values; (**d**) schematic illustration of the changes in the chemical structure of CR–PBz–FbM at different pH values. Reproduced with permission from [209]. Copyright 2016 The Royal Society of Chemistry.

Electrospinning has become an effective technique for synthesizing nanofibrous matrices because the surface-to-volume ratio and porous structures of nanofibrous matrices can be easily controlled [122,204]. In particular, electrospun fibers have become a promising template for oil/water separation because of their high specific surface areas and nanoscale pore structures [205]. There are several studies examining nanofiber membranes fabricated by electrospinning and their application in oil/water separation. A flexible carbon–SiO_2_ composite nanofibrous membrane was reported for oil/water separation [23]. An interpenetrating network consisting of carbon and SiO_2_ contributed to the improved properties of nanofibrous membranes. The nanofiber membranes exhibited excellent mechanical properties and good flexibility. Their wetting properties were sustained even at elevated temperatures, indicating excellent thermal and chemical stability. These membranes, which were coated with silicone oil, showed hydrophobic and superoleophilic characteristics, with the WCA and oil CA of 144° and 0°, respectively. Ma et al. reported cellulose acetate/polyimide electrospun nanofibrous membranes for oil/water separation [206]. These nanofiber membranes were further coated by a polybenzoxazine layer containing SiO_2_-NPs, which enhanced the mechanical strength of fibers. These nanofibrous membranes, with a high WCA of 160° and oil CA of 0°, exhibited tensile strengths higher than 200 MPa. The nanofibrous membranes performed rapid and effective oil/water separation for various oil/water mixtures. Alayande’s group fabricated electrospun PS and PS zeolite fibers with superhydrophobic and superoleophilic properties for crude oil/water separation [207]. Zeolite was embedded into the polymer matrix to impart a rough surface of electrospun nanofibers. These electrospun nanofibers showed superhydrophobic and superoleophilic characteristics with a WCA of 150° and crude oil CA of 0°, respectively, and performed efficient oil/water separation. Ning et al. fabricated fibrous membranes by the polymerization of styrene and butyl acrylate for oil/water separation [208]. The fibrous membranes with a WCA of 155° showed superhydrophobicity and lipophilicity and possessed a good capability for separating oil from water. pH-responsive electrospun-fibrous membranes were also prepared by electrospinning (Figure 11) [209]. The membrane exhibited a water to oil selective behavior at pH = 1, while it showed the opposite behavior at pH = 14, which demonstrated the completely reversible behavior due to a change in the pH of the solution (Figure 11a,b). A basic solution (pH = 14)-treated membrane turned the membrane from oil-selective to water-selective. The basic solution-treated membrane was water permeable and resistant to organic solvent permeation, led to a high-water permeation flux of 14,950 L/m^2^h on toluene–water separation. A hydrophobic overlapping bundle of fibers was created by thermally crosslinking polybenzoxazine (Figure 11c,d). However, the selectivity was not enough to be an excellent smart surface. The aforementioned electrospun-fibrous membranes have shown great potential for oil/water separation. However, significant challenges remain for large-scale industrial applications. Since the surface structures of electrospun nanofibers are easily damaged and fragile, the synthesis of stable and durable electrospun-fibrous membranes is a substantial challenge for securing resistance to acid and alkali corrosion. The poor mechanical stability of electrospun nanofibers is a major drawback for industrial applications as well. Most of the current study focuses on the design, synthesis, and performance of various materials for oil/water separation. Research on understanding the separation phenomena and control during oil/water separation and collection processes is necessary.

#### 3.3.2. Absorption-Based Materials

The aforementioned 2D superhydrophobic or underwater superoleophobic materials, such as meshes, fabrics, and membranes, have been utilized in various industrial settings, while they are less practical in cases of outside environmental oil spillage and pollution. These 2D filtering methods have difficulty removing or purifying oil-polluted water in special cases, such as oil being leaked onto the sea surface or heavy oils leaked onto the seafloor. These special cases can be addressed by 3D bulk materials, such as sponges, aerogels, and forms, because they do not suffer from the disadvantages of 2D bulk materials. 3D bulk materials can simply be placed on contaminated sites, and they will selectively absorb oil or water. Furthermore, there is no expensive or specialized equipment required for the quick removal of pollutants. In this section, we will discuss the technologies reported for quick, efficient, and cost-effective oil/water separation.

##### General Absorbents

**Figure 12:**
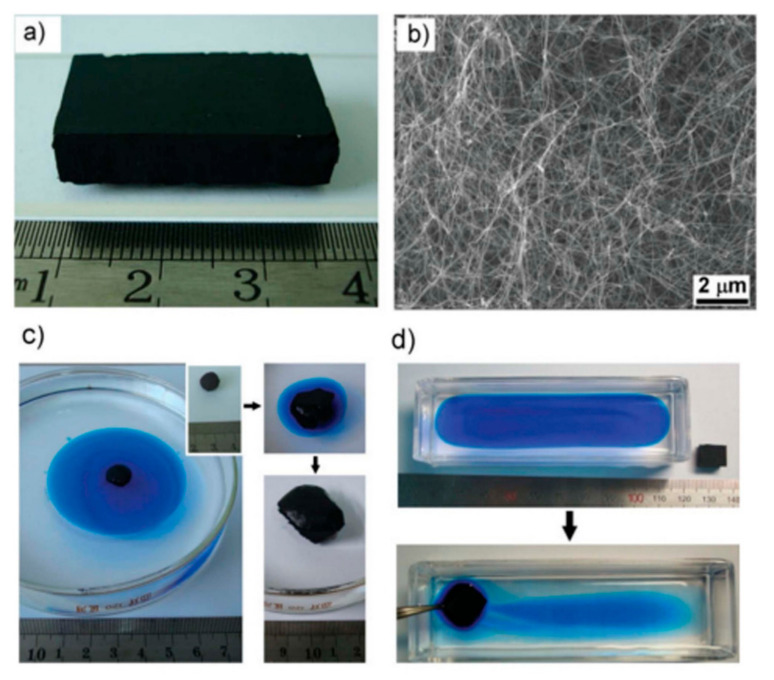
(**a**) A monolithic sponge with a size of 9.6 cm^3^; (**b**) cross-sectional SEM image of the sponge demonstrating a porous morphology and overlapped CNTs. Images showing the absorption of (**c**) an oil dyed with Oil Blue and (**d**) an oil strip distributed on water by a sponge. Reproduced with permission from [210]. Copyright 2010 Wiley-VCH Verlag GmbH & Co. KGaA, Weinheim.

Superhydrophobic and superoleophilic 3D porous bulk materials can selectively absorb the oil phase in an oil/water mixture when they contact the oil/water mixture. However, the water phase is repelled by 3D porous bulk materials due to their superhydrophobic surfaces. Only the oil phase is selectively absorbed by the 3D porous bulk materials [210,211,212,213,214,215]. The absorbed oil can be easily released from the 3D bulk materials for reuse by squeezing the 3D bulk materials. Superhydrophilic and underoil superoleophobic 3D porous bulk materials can selectively absorb the water phase in an oil/water mixture when they contact the oil/water mixture [170]. However, the oil phase is repelled by 3D porous bulk materials due to their superhydrophilic surfaces. Cui et al. fabricated a sponge-like 3D bulk material composed of interconnected carbon nanotube skeletons (Figure 12a,b) [210]. The sponge showed superhydrophobicity, high flexibility, very low density, and high porosity (>99%). The sponge could selectively absorb a vegetable oil film distributed on a water bath and continuous oil strip distributed in a rectangular water bath with significant selectivity (Figure 12c,d). De Luca’s group also fabricated multi-walled CNT absorbents for the removal of unleaded gasoline from water [211]. They achieved a high absorption capacity in a short absorption time using small amounts of CNT absorbents (0.7 g).

The absorption capacity of the sponge reached several hundred times the weight of the pristine sponge because of the swelling property of the sponge upon contact with liquids. The sponge could potentially be applied in spill cleanup by removing the spreading oil on a water surface. Zhu et al. synthesized a superhydrophobic and superoleophilic polyurethane (PU) sponge by a successive immersion process [212]. The superhydrophobic PU sponge could selectively absorb oils but repel water when the PU sponge was immersed into an oil/water mixture. By removing the sponge from the oil/water mixture, the oils could be easily removed from the oil/mixture. By squeezing the sponge, the sponge can easily release the absorbed oils. A superhydrophobic PU sponge was fabricated through an ultrasonic dip coating process [213]. The PU sponge effectively absorbed a wide range of oils floated on hot water or corrosive aqueous solutions. As a result, the oils were completely removed from the water surface. Gao et al. fabricated a carbon soot-coated MF sponge for the cost-effective oil absorption [214]. The carbon soot, obtained from a combustion flame, was coated onto the MF sponge by a simple dip-coating method. The absorbent could selectively eliminate oil contaminants from the oil/water mixture by an absorption process. A superhydrophobic PDMS sponge was prepared by the polymerization of a prepolymer and a curing agent [215]. NaCl particles were employed as the template. The PDMS sponge exhibited high absorption capacity and chemical/thermal stability. The PDMS sponge with special wettability was able to selectively absorb the oils either floating on the water surface or underwater. Hou et al. synthesized micro/nanoscale aggregates of poly((3,3,3-trifluoropropyl)methylsiloxane) through phase separation technology [216]. Superhydrophobic 3D porous materials were formed by the simple coating of the aggregates onto different porous 3D substrates. The superhydrophobic 3D porous materials could absorb oils from oil/water mixtures while repelling water. Du et al. synthesized a magnetic melamine foam with multidimensional and well defined surface structures through the precipitation and annealing method [217]. The superhydrophobic/superoleophilic magnetic melamine foam possessed a magnetic-driven property. The superoleophilic magnetic melamine foam could achieve remote-controlled oil/water separation. Song et al. fabricated a floating-oil collection prototype 3D device by combining different 2D meshes [186]. This mesh was installed on a container. The container, equipped with meshes, was partly dipped into an oil/water mixture. The floating oil could wet and pass through the mesh and enter and be collected by the container. However, the water was remained outside of the prototype 3D device. Aerogel, a porous ultralight material derived from a gel, can be used for absorption-based materials. Zou’s group synthesized polysiloxane aerogels for oil/water separation [218]. The polysiloxane aerogels with excellent physical properties were prepared by an eco-friendly synthetic method at supercritical CO_2_. This superhydrophobic aerogel absorbed 4.7–14.5 g g-1 of various organic solvents and showed excellent recyclability up to 100 cycles without a performance reduction. Yu’s group also reported the synthesis of sponge-like aerogels [219]. The aerogels were synthesized by using two different types of silanes in the presence of the hexadecyltrimethylammonium bromide. These hydrophobic gels showed an absorption capacity over 6 g g-1 for various organic solvents up to 10 cycles.

##### Multifunctional Absorbents

**Figure 13:**
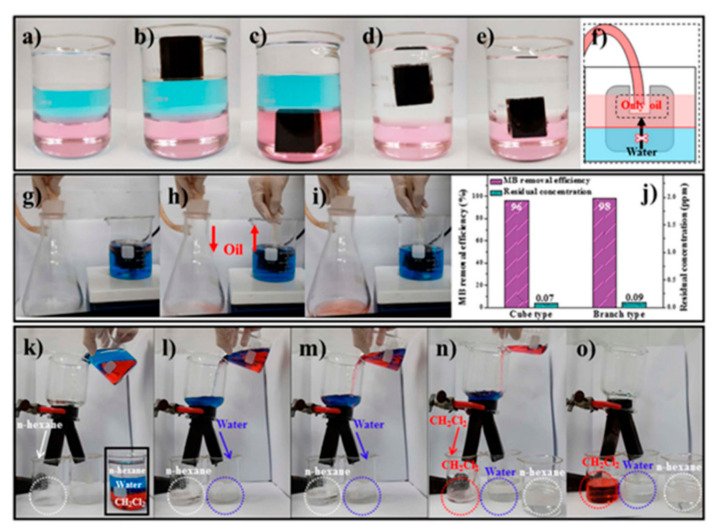
(**a**) Multiphase solutions containing 3 types of liquids such as n-hexane, water, and CCl_4_. The hydrophobic sponge stayed in the (**b**) n-hexane and (**c**) CCl_4_ layers. The anti-overturn Janus sponges (AJSs) remained at the (**d**) n-hexane/water and (**e**) water/CCl_4_ interfaces; (**f**–**i**) an AJS-based oil/water separation under artificial high waves; (**j**) removal efficiency and residual concentration of MB adsorbed by cube and AJSs; (**k**–**o**) successive oil/water separation and aqueous pollutant purification by a pant-like AJS. Reproduced with permission from [220]. Copyright 2018 The Royal Society of Chemistry.

Superhydrophobic and superhydrophilic sponges can be utilized to purify aqueous pollutants during the continuous separation of three multiphase solutions (light oil/water/heavy oil). Choi et al. developed an anti-overturn Janus sponge (AJS) with amphiprotic characteristics, such as hydrophobic and hydrophilic parts (Figure 13) [220]. The AJS can float or can be submerged on or under water, respectively, because of its amphiprotic property. The AJS can be synthesized by the successive coating of magnetic NPs (MNPs), Pdop, and ODA on an MF sponge. The hydrophobic ODA was coated only onto the upper half of the AJS, leading to the formation of an AJS consisting of hydrophilic (bottom) and hydrophobic (top) parts. The hydrophilic part remained submerged under water, while the hydrophobic part always floated on water. The AJS exhibited excellent adsorption characteristics for aqueous pollutants during fast oil/water separation because the AJS also possessed a hydrophilic part (MFS/MNP/Pdop). The AJS can remain at each interface in three multiphase solutions and adsorb aqueous pollutants due to its amphiprotic property (Figure 13a–e). The AJS rapidly separated an oil/water mixture using a pump as well (Figure 13f–j). A pant-like structure that was divided into two branches could continuously separate an oil/water mixture (light oil/water/heavy oil) and rapidly purify contaminated water during the separation (Figure 13k–o).

**Figure 14:**
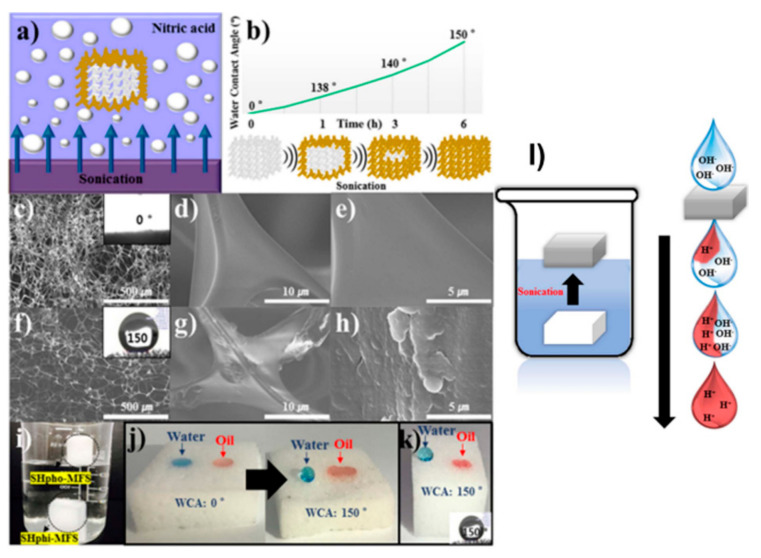
(**a**) Schematic illustration for the switching of the superhydrophilic-MFS to the superhydrophobic-MFS and (**b**) the wettability changes of the superhydrophilic-MFS; (**c**–**h**) SEM images of the superhydrophilic-MFS (**c**–**e**) before and (**f**–**h**) after ultrasound treatment; (**i**) image showing the superhydrophilic-MFS (**bottom**) before and (**top**) after ultrasound treatment; (**j**) Water contact angles (WCAs) and oil contact angle (OCAs) on the superhydrophilic-MFS (**left**) before and (**right**) after ultrasound treatment; (**k**) WCA and OCA on the inside cross-sectional surface of superhydrophilic-MFS after ultrasound treatment; (**l**) schematic illustration for tuning a strong base into a tailored pH solution by a simple filtration. Reproduced with permission from [221]. Copyright 2019 Wiley-VCH Verlag GmbH & Co. KGaA, Weinheim.

Superhydrophobic and superhydrophilic sponges could also be utilized as novel neutralizers during oil/water separation. The one-step switching of a superhydrophilic sponge to a superhydrophobic sponge with pH tuning ability was demonstrated by ultrasound treatment at a low pH (Figure 14a,b) [221]. After wettability switching, the superhydrophobic sponge could selectively separate both oil and water because of the proton release properties of the superhydrophobic sponge (Figure 14c–k). The compressed superhydrophobic sponge also showed excellent separation performance for a W/O emulsion. Furthermore, an aqueous solution of a strong base was neutralized or tuned into a certain solution with a tailored pH by a simple filtration step because the superhydrophobic sponge could produce protons in an aqueous solution during oil/water separation (water removal) (Figure 14l). The superhydrophobic sponge was also able to prevent a sudden reaction during neutralization.

**Figure 15:**
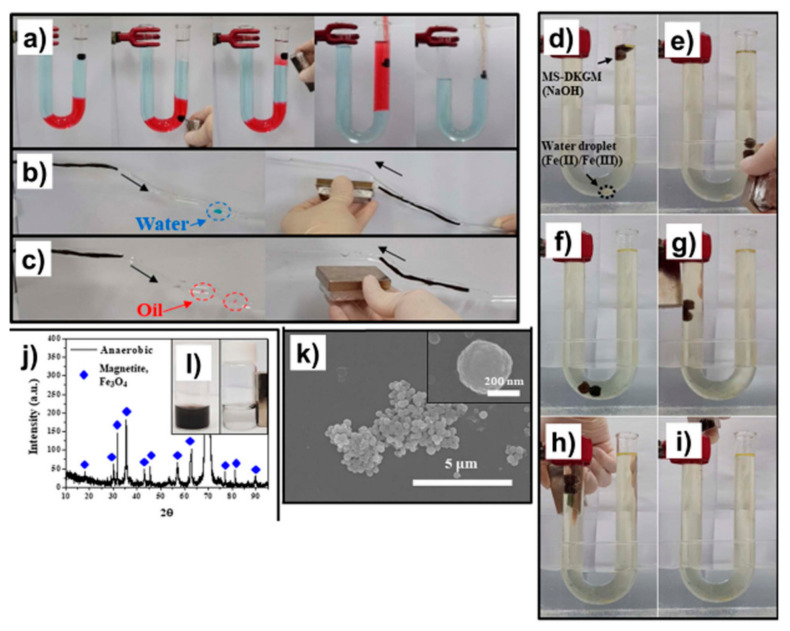
(**a**) Images showing the removal of heavy oils below the water layer in a U-shaped tube containing a multiphase solution composed of n-hexane (transparent), water (colored blue), and chloroform (colored red); (**b**) water or (**c**) chloroform droplets in hexane or water within a fine channel (diameter: 3 mm) using magnetic thread (MT)-DKGM (deacetylate konjac glucomannan) or MT-PDMS, respectively; (**d**–**i**) images showing the anaerobic reaction systems for the synthesis of magnetite NPs using MS-DKGM; (**j**) XRD and (**k**) SEM data of the black solution sample collected from the MS-DKGM balls after the reaction; (**l**) black solution samples (**left**) before and (**right**) after applying a magnetic field. Reproduced with permission from [164]. Copyright 2019 American Chemical Society.

Most superhydrophobic and superhydrophilic absorbents have performed oil/water separation in places that the user can easily access. Oil/water separation in tubes, pipes, and tanks has not been reported because user access is limited. To address this issue, magnetic sponges (MSs) and magnetic threads (MTs) were synthesized. They could perform remote-controlled oil/water separation in tubes, pipes, and tanks that user access is limited (Figure 15) [164]. Konjac glucomannan (KGM)-coated MS, a hydrophilic MS, was synthesized by a mixture coating process of MNPs and KGM on an MF sponge. PDMS-coated MS, a hydrophobic MS, was also prepared by further coating PDMS onto a hydrophilic MS, and scattered oil or water droplets in water or oil within a tube were completely eliminated by the movement of the hydrophobic MS or hydrophilic MS sample, respectively (Figure 15a). The hydrophilic or hydrophobic MT also performed magnetic field-driven oil/water separations within a fine channel (Figure 15b,c). By manipulation of an external magnetic field, a hydrophobic MS spherical sponge was moved to the heavy oil, absorbed the heavy oil below the water, returned to the light oil layer, and released the heavy oil (Figure 15a). After the heavy oil was delivered, the mixed oil consisting of light and heavy oils was completely eliminated by a suction pump. Since the MS possessed the porous structure, the strong magnetic property, the prewetting ability, and the oil layer, the MS could be used for anaerobic reaction systems as well (Figure 15d–k).

## 4. Summary and Perspective

In this review, we briefly summarized the recent trends of air filtration and oil/water separation technologies using 2D and 3D bulk materials, including meshes, fabrics, membranes, sponges, and foams. These 2D and 3D materials have shown outstanding performances in air filtration and selective oil/water separation. However, technical problems or challenges still need to be addressed in future studies. For air filtration, there are two general issues: removal efficiency and pressure drop. Although most reported air filters can remove PM_2.5_ particles, a diverse range of air filters for capturing PM particles smaller than PM_2.5_ particles should be developed because smaller particles can penetrate blood vessels and travel through the circulatory system. Air filtration design to remove smaller PM particles should be emphasized in the future. To achieve such a goal, the material and structure of air filters deserve more attention. Air filter materials possessing good affinity with PM particles should be developed. Relatively little attention has been focused on hydrophobic materials and their roles. Studying air filters possessing the same structure but different surface wettability, such as hydrophilic, hydrophobic, and hydrophilic/hydrophobic hybrid filters, would be helpful for understanding the role of hydrophobicity and determining an optimized air filter material. Air filters with hierarchical structures are also needed for the removal of smaller PM particles. However, enhancing performance with dense or complicated filter structures leads to a detrimentally high pressure drop. The development of air filters as alternatives to typical filter structures might decrease the pressure drop. The study of the structural transformation or modification of air filters would provide new possibilities for advanced air filtration.

For oil/water separation, there are several challenges. First, most reported materials have relatively poor mechanical and chemical durability because these materials have hierarchically rough surface morphologies when coated with hydrophobic materials. These fragile surface structures are easily damaged, leading to an irreversible decline in oil/water separation performance. Thus, a study to address this issue is necessary. Second, the oil pollutants in a real situation are usually very complex rather than the pure oils that are used in the laboratory. Oil pollutants in oil leakage accidents are a mixture of different components of floating matter, oil, solvent, acid, base, surfactants and so on. Particularly, the pores of oil/water separators can be easily clogged by floating matter in oceans before oil/water separation can occur, leading to a failure of the oil/water separation process. Third, laboratory separation systems can only separate small amounts of oil/water mixtures, which is not suitable for oil spill accident places where large amounts of oily wastewater need to be separated. Finally, the separation technologies of O/W and W/O emulsions are also necessary because these emulsions account for considerable amounts of oily wastewaters. Membranes might be good candidates because they show a relatively high performance due to their small pore size. However, they also suffer from lower separation capacity (flux) because of their small pore size. Despite the intensive studies in the fields of air filtration and oil/water separation in recent years, important challenges remain. The aforementioned applications are based on the nanomaterial-loaded 2D and 3D bulk materials. Thus, the development of the following issues is needed to address the aforementioned technical problems and challenges: (i) more advanced 2D and 3D bulk materials loaded with nanomaterials, (ii) nanomaterial-leakage prevention technology, (iii) environmentally friendly materials, and (iv) a new field of application.

## 5. Conclusions

In this article, we provided a summary of recent progress in 2D and 3D bulk material-based air filtration and oil/water separation. The concepts, backgrounds, employed materials, advantages, fabrication methods, and characteristics of air filters and oil/water separators were discussed in each section. We ended the review with the challenges, technical problems, and future research directions. Since the aforementioned application fields are based on the knowledge and technique from different research fields, such as chemistry, physics, biology, polymer, nanomaterial, and their composites, multidisciplinary studies are highly needed for advanced air filtration and oil/water separation.

## Figures and Tables

**Table 1:** Typical 2D and 3D bulk materials for air filters and oil/water separators.

Application Type	2D	3D
Air filter	Meshes Fiber nets Fabrics Papers	Sponges Sponge/polymer networks Sponge/paper networks
Oil/water separator	Meshes Membranes Fabrics	Sponges Forms Aerogels Rubber networks
